# The impact of dietary fiber on colorectal cancer patients based on machine learning

**DOI:** 10.3389/fnut.2025.1508562

**Published:** 2025-01-24

**Authors:** Xinwei Ji, Lixin Wang, Pengbo Luan, Jingru Liang, Weicai Cheng

**Affiliations:** Department of Gastrointestinal Surgery, Yantaishan Hospital, Yantai, China

**Keywords:** colorectal cancer, dietary fiber, enteral nutrition support, nutritional status, machine learning

## Abstract

**Objective:**

This study aimed to evaluate the impact of enteral nutrition with dietary fiber on patients undergoing laparoscopic colorectal cancer (CRC) surgery.

**Methods:**

Between January 2023 and August 2024, 164 CRC patients were randomly assigned to two groups at our hospital. The control group received standard nutritional intervention, while the observation group received enteral nutritional support containing dietary fiber. Both groups were subjected to intervention and continuously observed until the 14th postoperative day. An observational analysis assessed the impact of dietary fiber intake on postoperative nutritional status in CRC patients. The study compared infection stress index, inflammatory factors, nutritional status, intestinal function recovery, and complication incidence between groups. Additionally, four machine learning models—Logistic Regression (LR), Random Forest (RF), Neural Network (NN), and Support Vector Machine (SVM)—were developed based on nutritional and clinical indicators.

**Results:**

In the observation group, levels of procalcitonin (PCT), beta-endorphin (*β*-EP), C-reactive protein (CRP), interleukin-1 (IL-1), interleukin-8 (IL-8), and tumor necrosis factor-alpha (TNF-*α*) were significantly lower compared to the control group (*p* < 0.01). Conversely, levels of albumin (ALB), hemoglobin (HB), transferrin (TRF), and prealbumin (PAB) in the observation group were significantly higher than those in the control group (*p* < 0.01). Furthermore, LR, RF, NN, and SVM models can effectively predict the effects of dietary fiber on the immune function and inflammatory response of postoperative CRC patients, with the NN model performing the best. Through the screening of machine learning models, four key predictors for CRC patients were identified: PCT, PAB, ALB, and IL-1.

**Conclusion:**

Postoperative dietary fiber administration in colorectal cancer enhances immune function, reduces disease-related inflammation, and inhibits tumor proliferation. Machine learning-based CRC prediction models hold clinical value.

## 1 Introduction

Colorectal cancer (CRC) ranks as the third most prevalent malignancy worldwide, and its incidence rate is on the rise (1). In 2018, there were 18.1 million new cancer cases globally, with CRC ranking fourth among them. Given this prevalence, understanding the pathogenesis of CRC and developing practical treatment approaches is crucial (2, 3). The development of CRC is complex, involving genetic and environmental factors that work together to promote abnormal growth in colorectal tissue, leading to tumor formation. Environmental factors, particularly dietary habits, exposure to radiation, and environmental toxins, significantly influence CRC development. High-calorie, high-fat diets, coupled with disruptions in intestinal microbiota and local inflammation, are critical factors in initiating CRC (4).

The impact of dietary factors on CRC is significant (5). Prolonged consumption of high-calorie, high-fat diets can disrupt the balance of intestinal bacteria and lead to local inflammation, creating an environment conducive to CRC development. Chronic inflammation resulting from prolonged synthesis of pro-inflammatory cytokines is a crucial contributor to autoimmune diseases and cancer (6). Addressing this inflammation and restoring immune balance is critical in preventing complications. In conclusion, given the global burden of CRC and its multifaceted etiology involving genetic and environmental factors, particularly dietary habits and inflammation, there is an urgent need to explore dietary interventions that can mitigate inflammation, prevent associated complications, and restore immune balance effectively (7).

In the context of postoperative care for CRC patients, the role of dietary fiber is pivotal due to its impact on intestinal health (8). Dietary fiber plays a crucial role in safeguarding the intestinal barrier, regulating immune function, and mitigating postoperative inflammatory reactions (9). Despite these benefits, limited research exists on the early integration of dietary fiber into CRC patient management post-surgery. Malnutrition not only hampers recovery but also heightens the risk of complications and mortality rates. Moreover, immune suppression could potentially enhance the chances of tumor metastasis and recurrence. Consequently, providing early nutritional support after CRC surgery is paramount. In recent years, the advancement of machine learning has led to the widespread application of algorithms such as Random Forest (RF), Logistic Regression (LR), Support Vector Machine (SVM), and Neural Network (NN) in clinical research (10). These algorithms facilitate the development of disease diagnosis and prediction models, thereby enhancing decision-making processes (11). This study aimed to investigate the effects of dietary fiber on postoperative immune function and inflammatory responses in CRC patients, identify critical factors influencing CRC, and offer valuable insights for the prevention and management of CRC.

## 2 Methodology

### 2.1 Materials and methods

We conducted a prospective randomized controlled trial on CRC patients undergoing surgical treatment, focusing on the effects of dietary fiber intervention on the patients. The study received written approval from our hospital’s review committee (Ethical Review No. 2023027), and all participating CRC patients provided informed consent. The trial procedures complied with clinical practice guidelines as well as the principles outlined in the Helsinki Declaration.

### 2.2 Patient selection

From January 2023 to August 2024, we selected 164 CRC patients admitted to our Department of Gastroenterology and randomly assigned them to control group and observation group, each consisting of 82 patients (Table 1). The observation group consisted of 46 males and 36 females, with a mean age of 54.9 ± 11.1 years. The control group included 44 males and 38 females, with a mean age of 56.2 ± 13.9 years. Comparison of general data between the two patient groups indicated that there were no statistically significant differences (*p* > 0.01). Notably, this study received approval from the Ethics Committee of the China Railway Center Hospital of China National Pharmaceutical Corporation, and informed consent was obtained from both patients and their families. All surgeries were conducted in accordance with the guiding principles established by the Helsinki Declaration.

**Table 1:** Characteristics of cases.

Variable	Observation group (*n* = 82)	Control group (*n* = 82)	*p*
Sex (%)			
Male	46 (56.1)	44 (53.7)	0.61
Female	36 (43.9)	38 (36.7)	0.57
Age mean (SD) year	54.9 ± 11.1	56.2 ± 13.9	0.66
Alcohol status (%)			
Drinkers	30 (36.6)	34 (41.5)	0.72
Nondrinkers	52 (63.4)	48 (58.5)	0.79
Smoke status (%)			
Smokers	34 (41.5)	36 (43.9)	0.67
No smokers	48 (58.5)	46 (56.1)	0.71
TNM stage (%)			
I and II	42 (51.2)	36 (43.9)	0.81
III	40 (48.8)	46 (56.1)	0.86

Observation group: using enteral nutrition support containing dietary fiber; Control group: using routine nutritional interventions.

Inclusion criteria: The diagnosis was confirmed by clinical symptoms and signs, endoscopic imaging, and laboratory tests in our hospital, according to the criteria of “Consensus opinion on diagnosis and treatment of inflammatory bowel disease diseases.” Truelove-witts score was used as a reference for the diagnosis of severity, and the diagnosis was confirmed by pathological examination.

Exclusion criteria: complicated with other types of organ dysfunction; complicated with intestinal fungal or viral infection; complicated with colon polyp; pregnant or lactating women; For various reasons caused by the lack of clinical data.

Observational indicators: In this study, we aim to compare various indicators between two groups of patients following CRC surgery. Specifically, we will analyze infection stress markers, inflammatory response factors, nutritional status, recovery of intestinal function, and the occurrence of complications in both groups.

Before and after the intervention, 5 mL of venous blood will be collected from each patient to assessed infection stress markers such as procalcitonin (PCT), *β*-endorphin (β-EP), and C-reactive protein (CRP). Additionally, we used the same method to measure inflammatory response factors in both groups, including interleukin-1 (IL-1), interleukin-8 (IL-8), and tumor necrosis factor-alpha (TNF-*α*). Before and after the intervention, 3 mL of fasting venous blood samples was drawn from each patient to evaluate parameters like hemoglobin (HB), albumin (ALB), transferrin (TRF), and prealbumin (PAB) to track changes in their health status. We assessed immune function through markers like cluster of differentiation 4 positive (CD4+), cluster of differentiation 3 positive (CD3+), cluster of differentiation 8 positive (CD8+), immunoglobulin A (IgA), immunoglobulin M (IgM), and immunoglobulin G (IgG) levels. Lastly, we recorded crucial postoperative indicators such as bowel sound recovery, the first appearance of anal gas passage, and the timing of the first defecation to track the progress of each patient’s recovery journey.

### 2.3 Methods

None of the patients had a gastrointestinal decompression tube inserted before surgery. The observation group and the control group were given continuous nutritional intervention for 14 days post-surgery. For the observation group, the nutritional intervention during postoperative days 1–7 included an intravenous drip of 500 mL of Rui Gao (Fresenius Kabi Huarui Pharmaceuticals Co., Ltd.), oral administration of 25 g of dietary fiber (Tiantian Yikang Biotechnology Co., Ltd., composed primarily of polyglucose, oligofructose, glycerol, citric acid, and potassium sorbate), and 4 g of Golden Bifidobacterium (Shuangqi Pharmaceutical Co., Ltd.). From postoperative days 8 to 14, the intravenous drip of Rui Gao was increased to 1,000 mL daily, while the oral doses of dietary fiber (25 g) and Golden Bifidobacterium (4 g) remained consistent. The infusion rate during days 1 to 7 post-surgery was set at 30 to 50 mL/h, which was then increased to 60 to 70 mL/h from days 8 to 14, adjusted according to each patient’s physiological tolerance. For the control group, nutritional interventions from postoperative days 1 to 7 included an intravenous drip of 500 mL of Rui Gao and oral administration of 4 g of Golden Bifidobacterium. From postoperative days 8 to 14, the intravenous drip of Rui Gao was increased to 1,000 mL daily, while the oral dose of Golden Bifidobacterium (4 g) remained the same. However, the method and infusion rate remained identical to those in the observation group. Both groups were continuously monitored and assessed over the 14 days following surgery.

### 2.4 Statistical analysis

Statistical analysis was carried out using SPSS 22.0 software in this observational study. Pairwise comparisons within the group were performed utilizing paired t-tests for continuous variables, which were depicted as mean ± standard deviation ( $\bar{x}$ ± s). Descriptive statistics were employed for continuous data, whereas paired t-tests were utilized for analysis. Categorical variables were characterized by their frequencies and percentages, and the analysis was conducted using the chi-square test. All cases were randomly divided into a training set (80%) and a testing set (20%). The NN model in this study includes input layer, hidden layer, and output layer. Among them, the input layer has 9 neurons, the hidden layer has 30 neurons, and the output layer has 1 neuron. The activation function was ReLU, and the output layer used the Sigmoid activation function. The machine learning models LR, RF, NN, and SVM in this study were developed using Python 3.10. The performance of all models was assessed using the area under the curve (AUC), accuracy, sensitivity, specificity, and F1 score metrics. A significance level of *p* < 0.01 denoted statistical significance throughout the analysis.

## 3 Results

### 3.1 Comparison of infection stress indexes

The infection stress indicators (PCT, *β*-EP, and CRP) of each group in POD-7 and POD-14 were significantly reduced compared to POP levels. Although no significant differences were found in the levels of PCT, *β*-EP, and CRP between the groups at different time points (*p* > 0.01), it is important to note that, at two specific time points, PCT, β-EP, and CRP levels in the observation group were significantly lower than those in the control group (*p* < 0.01), as shown in Table 2.

**Table 2:** Comparison of infection stress indexes between the two groups.

Variable	POP	POD-7	POD-14
PCT (μg/L)			
Control group	6.62 ± 1.33	2.21 ± 0.31	1.55 ± 0.23
Observation group	6.79 ± 1.26	2.01 ± 0.21^*^	1.39 ± 0.19^*^
Mean difference	6.71 ± 1.29	2.11 ± 0.26	1.47 ± 0.21
*t*	0.117	1.883	3.780
*p*	0.611	<0.01	<0.01
β-EP (ng/L)			
Control group	71.55 ± 5.33	52.31 ± 4.69	46.37 ± 4.44
Observation group	71.32 ± 5.16	50.12 ± 4.71^*^	39.77 ± 4.16^*^
Mean difference	71.44 ± 5.25	51.22 ± 4.70	43.07 ± 4.30
t	0.073	7.61	9.19
*p*	0.761	<0.01	<0.01
CRP (ng/L)			
Control group	6.62 ± 1.33	2.21 ± 0.31	1.55 ± 0.23
Observation group	6.79 ± 1.26	2.01 ± 0.21^*^	1.39 ± 0.19^*^
Mean difference	6.71 ± 1.29	2.11 ± 0.26	1.47 ± 0.21
t	0.065	11.66	13.11
*p*	0.854	<0.01	<0.01

POP, pre-operation; POD, postoperative day; PCT, procalcitonin; β-EP, β-endorphin; CRP, C-reactive protein; t, *t*-test compared to the observation group; ^*^p < 0.01, compared with the observation group.

### 3.2 Comparison of inflammatory response factors

The levels of IL-1, IL-8, and TNF-*α* in both groups gradually decreased at POD-7 and POD-14, and the differences in the levels of inflammatory response factors within the groups at each time point were not statistically significant (*p* > 0.01). The levels of IL-1, IL-8, and TNF-α in the observation group at POD-14 were significantly lower than those in the control group (*p* < 0.01), as shown in Table 3.

**Table 3:** Comparison of inflammatory response factors between the two groups.

Variable	POP	POD-7	POD-14
IL-1			
Control group	59.96 ± 5.06	51.63 ± 4.11	46.11 ± 3.33
Observation group	60.31 ± 5.13	45.39 ± 3.96^*^	30.97 ± 3.67^*^
Mean difference	60.12 ± 5.10	48.51 ± 4.04	38.54 ± 3.50
t	0.029	9.33	20.181
*p*	0.773	<0.01	<0.01
IL-8			
Control group	61.16 ± 4.96	44.16 ± 4.41	38.93 ± 4.11
Observation group	61.11 ± 5.01	29.16 ± 4.33	20.87 ± 3.91^*^
Mean difference	61.14 ± 4.99	36.66 ± 4.37	29.90 ± 4.01
t	0.049	17.36	19.117
*p*	0.962	0.09	<0.01
TNF-α			
Control group	37.88 ± 3.24	27.03 ± 3.11	17.15 ± 2.76
Observation group	37.85 ± 3.21	19.61 ± 2.73^*^	13.54 ± 2.21^*^
Mean difference	37.87 ± 3.23	23.32 ± 2.92	15.35 ± 2.49
t	0.071	5.73	7.331
*p*	0.964	<0.01	<0.01

POP, pre-operation; POD, postoperative day; IL-1, interleukin-1; IL-8, interleukin-8; TNF-α, tumor necrosis factor-alpha; t, *t*-test compared to the observation group; ^*^p < 0.01, compared with the observation group.

### 3.3 Comparison of nutritional status

**Figure 1:**
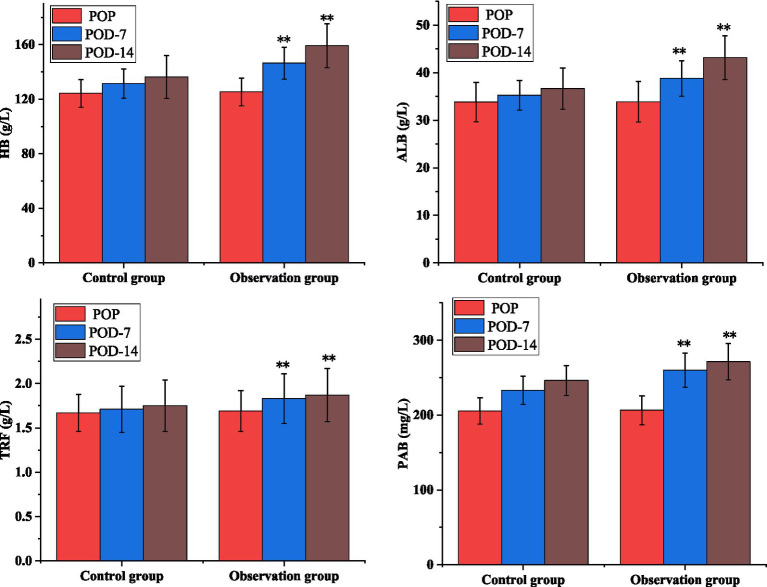
Comparison of nutritional status of patients.

As shown in Table 4, the levels of nutritional factors: HB, ALB, PAB, and TRF increased significantly between the two groups at POD-7 and POD-14 at each time points, but the differences in the levels of each nutritional indicator within the groups were not statistically significant (*p* > 0.01). As shown in Figure 1, the nutritional parameters HB, ALB, PAB, and TRF were significantly elevated in the POD-14 observation groups compared to the control group (*p* < 0.01).

**Table 4:** Comparison of nutritional status between the two groups.

Variable	POP	POD-7	POD-14
HB (g/L)			
Control group	124.26 ± 10.17	131.46 ± 10.77	136.23 ± 15.73
Observation group	125.37 ± 10.22	146.31 ± 11.63^*^	159.16 ± 16.12^*^
Mean difference	124.82 ± 10.20	138.89 ± 11.20	147.70 ± 15.93
t	0.547	3.23	6.71
*p*	0.581	<0.01	<0.01
ALB (g/L)			
Control group	33.81 ± 4.13	35.23 ± 3.11	36.65 ± 4.31
Observation group	33.89 ± 4.27	38.81 ± 3.75^*^	43.19 ± 4.63^*^
Mean difference	33.85 ± 4.20	37.02 ± 3.43	39.92 ± 4.47
t	0.178	5.103	6.871
*p*	0.769	<0.01	<0.01
TRF (g/L)			
Control group	1.67 ± 0.21	1.71 ± 0.26	1.75 ± 0.29
Observation group	1.69 ± 0.23	1.83 ± 0.28	1.87 ± 0.30^*^
Mean difference	1.68 ± 0.22	1.77 ± 0.27	1.81 ± 0.30
t	0.046	0.863	1.712
*p*	0.901	0.06	<0.01
PAB (mg/L)			
Control group	205.37 ± 17.43	233.08 ± 18.67	246.13 ± 20.11
Observation group	206.41 ± 18.31	259.89 ± 22.77^*^	271.33 ± 24.32^*^
Mean difference	205.89 ± 17.87	246.49 ± 20.72	258.73 ± 22.22
t	0.438	3.111	5.901
*p*	0.836	<0.01	<0.01

POP, pre-operation; POD, postoperative day; HB, Hemoglobin; ALB, albumin; PAB, pre-albumin; TRF, transferring; t, *t*-test compared to the observation group; ^*^p < 0.01, compared with the observation group.

### 3.4 Comparison of intestinal function recovery

As shown in Table 5, compared with before intervention, the recovery of intestinal function in the observation group on the 7th and 14th days after intervention was better than the control group (*p* < 0.01).

**Table 5:** Comparative analysis of intestinal function between the two groups.

Variable	Recovery of intestinal sounds (h)	First anal exhaust (h)	First bowel movement (h)
Control group	34.26 ± 3.57	59.89 ± 5.11	91.23 ± 11.03
Observation group	25.31 ± 3.36^*^	47.74 ± 5.23^*^	71.22 ± 8.62^*^
t	11.101	9.76	10.37
*p*	<0.01	<0.01	<0.01

t, *t*-test compared to the observation group; ^*^p < 0.01, compared with the observation group.

### 3.5 Comparison of immune function

The levels of CD3+, CD4+, CD4+ / CD8+, IgA, IgM, and IgG in both groups of patients significantly decreased on POD-7 and gradually increased on POD-14. However, the differences in immune indicator levels between the two groups at each time point were not statistically significant (*p* > 0.01). As depicted in Table 6, after 7 days of dietary fiber support post-surgery, the levels of immune indicators in the observation group were significantly elevated compared to those in the control group (*p <* 0.01).

**Table 6:** Comparative analysis of immune function between the two groups.

Variable	POP	POD-7	POD-14
CD3+ (%)			
Control group	53.47 ± 5.17	49.77 ± 6.33	51.88 ± 6.93
Observation group	54.03 ± 6.52	51.21 ± 7.03^*^	56.11 ± 7.43^*^
Mean difference	53.75 ± 5.85	50.49 ± 6.68	53.99 ± 7.18
t	0.147	4.697	6.382
*p*	0.761	<0.01	<0.01
CD4+/CD8+			
Control group	0.91 ± 0.37	0.89 ± 0.39	1.07 ± 0.57
Observation group	0.84 ± 0.41	1.01 ± 0.43^*^	1.31 ± 0.61^*^
Mean difference	0.88 ± 0.39	0.95 ± 0.41	1.19 ± 0.59
t	0.009	3.94	4.23
*p*	0.823	<0.01	<0.01
CD4+ (%)			
Control group	32.49 ± 4.32	34.23 ± 5.11	35.15 ± 6.21
Observation group	32.10 ± 4.13	34.91 ± 5.77^*^	39.21 ± 6.67^*^
Mean difference	32.29 ± 4.23	34.57 ± 5.44	37.18 ± 6.44
t	0.113	4.891	6.151
*p*	0.969	<0.01	<0.01
IgM (g/L)			
Control group	1.71 ± 0.37	1.51 ± 0.31	1.95 ± 0.76
Observation group	1.74 ± 0.31	2.27 ± 0.47^*^	2.51 ± 0.55^*^
Mean difference	1.73 ± 0.34	1.89 ± 0.39	2.23 ± 0.66
t	0.055	13.45	13.78
*p*	0.901	<0.01	<0.01
IgA (g/L)			
Control group	1.63 ± 0.41	1.81 ± 0.62	2.53 ± 0.60
Observation group	1.66 ± 0.37	2.27 ± 0.57^*^	3.19 ± 0.69^*^
Mean difference	1.65 ± 0.39	2.04 ± 0.59	2.86 ± 0.65
t	0.038	3.31	5.99
*p*	0.76	<0.01	<0.01
IgG (g/L)			
Control group	10.11 ± 3.27	7.21 ± 2.56	9.03 ± 7.78
Observation group	9.43 ± 2.73	8.39 ± 2.27^*^	10.93 ± 3.53^*^
Mean difference	9.77 ± 3.0	7.8 ± 2.41	9.98 ± 5.66
t	0.072	2.34	2.97
*p*	0.501	<0.01	<0.01

POP, pre-operation; POD, postoperative day; CD3+, cluster of differentiation 3 positive; CD4+, cluster of differentiation 4 positive; CD8+, cluster of differentiation 8 positive; IgA, immunoglobulin A; IgM, immunoglobulin M; IgG, immunoglobulin G; t, *t*-test compared to the observation group; ^*^p < 0.01, compared with the observation group.

### 3.6 Model training process

To develop and evaluate classification models, we employed four commonly used machine learning algorithms: NN, SVM, LR, and RF. Additionally, we combined these classification models using ensemble and cascaded methods to assess the performance of the integrated classifier.

### 3.7 Assessment outcomes of various machine learning algorithms

**Figure 2:**
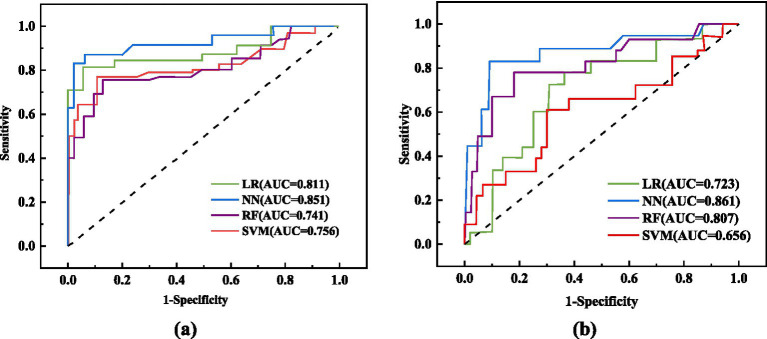
ROC curve analysis of different models. **(A)** ROC curves of 4 machine learning models in the training set; **(B)** ROC curves of 4 machine learning models in the test set.

This study assessed the performance of four machine learning algorithms by employing five essential evaluation metrics: AUC, F1, specificity, accuracy, and sensitivity score, as shown in Table 7. We plotted ROC curves for models built with the training and testing sets (Figure 2). The NN model distinguished itself as the best performer among the models. In the training set, the NN model achieved an AUC of 0.851, and in the testing set, it reached an AUC of 0.861. In comparison, the AUC values for the LR, RF, and SVM models were significantly lower. Given the superior performance demonstrated by the NN model, we ultimately chose it as the final model for this investigation.

**Table 7:** The efficacy of various machine learning algorithm models.

	AUC	Accuracy	Sensitivity	Specificity	F1
LR					
Train	0.811	0.801	0.723	0.841	0.737
Test	0.723	0.711	0.663	0.857	0.681
NN					
Train	0.851	0.811	0.689	0.831	0.724
Test	0.861	0.828	0.716	0.837	0.767
RF					
Train	0.741	0.788	0.632	0.743	0.661
Test	0.807	0.796	0.593	0.821	0.613
SVM					
Train	0.756	0.721	0.754	0.756	0.673
Test	0.656	0.746	0.704	0.803	0.676

LR, Logistic regression; NN, Neural network; RF, Random forest; SVM, Support vector machine.

### 3.8 Predictors of model

**Figure 3:**
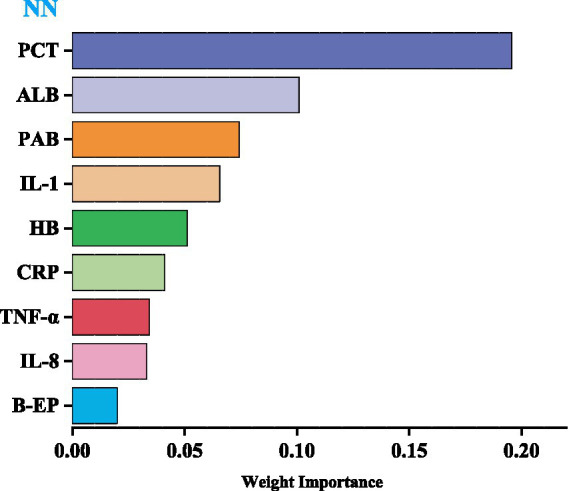
Importance of predictors for NN model.

We utilized the top-performing NN model to visualize the importance of nine variables based on their respective weights. The length of each bar in the chart is proportional to the significance of each variable. The variables refined by the NN model were then incorporated into a Logistic regression model, employing a stepwise regression method, ultimately retaining four critical influencing factors: PCT, PAB, ALB, and IL-1, as shown in Table 8 (Figure 3).

**Table 8:** Factors influencing the screening characteristics of logistic regression analysis.

Variable	*β*	*OR*	95%*CI*	*p*	*SE*	*Wald χ^2^*
PCT	0.019	1.011	1.001–1.016	0.001	0.002	8.116
PAB	0.146	1.122	1.046–1.197	0.003	0.037	7.343
ALB	−1.103	0.287	0.071–0.501	0.000	0.111	65.167
IL-1	0.216	1.206	1.097–1.311	0.001	0.061	10.89
Intercept	−6.597	0.003	0.000–7.161	0.102	4.151	2.316

PCT, procalcitonin; PAB, prealbumin; ALB, albumin; IL-1, interleukin-1.

## 4 Discussion

This study analyzed the impact of dietary fiber intake on the postoperative nutritional status of CRC patients. The levels of PCT, *β*-EP, CRP, IL-1, IL-8, and TNF-*α* in the observation group were significantly lower compared to those in the control group (*p* < 0.01). Furthermore, we developed LR, RF, NN, and SVM models, which demonstrated excellent performance in predicting and diagnosing CRC. The NN model outperformed the others, achieving an AUC of 0.851 in training and 0.861 in testing. PCT, PAB, ALB, and IL-1 are key factors for predicting CRC patients.

CRC is a common malignant disease that impacts the digestive system and is frequently managed through laparoscopic surgery to improve patient health and prolong survival (12). Although malnutrition is common among CRC patients, originating from the disease and surgical procedures, as well as the possibility of exacerbation by anesthetic drugs and stress responses during surgery, addressing these concerns is vital (13, 14). Due to the substantial malnutrition rates in CRC patients stemming from the disease and surgical impacts, compounded by possible exacerbation by anesthesia and surgical stress responses, addressing these issues directly is paramount. Changes in the gut microbiota, influenced by factors like surgical anesthesia and surgical stress responses, may result in dysbiosis and worsen malnutrition, increasing the risk of postoperative infections and inflammation, which can hinder patient recovery and long-term outlook (15).

Implementing scientifically proven nutritional support strategies is crucial to improve postoperative nutritional wellbeing in patients (16). These interventions include preoperative nutritional assessments, perioperative nutritional support, and the utilization of appropriate diets and intestinal nutritional supplements. Tailored nutritional plans should be created according to the individual conditions and dietary requirements of each patient. Nutritional improvements can be achieved through dietary modifications, oral supplements, and other appropriate approaches before surgery. Enteral or parenteral nutritional support can be administered during and after surgery to fulfill the patient’s energy, protein, and other dietary needs. Vigilant monitoring of patients’ nutritional statuses and relevant indicators is essential to adjust intervention plans as needed in a timely.

Early intestinal nutritional support is crucial for accelerating gastrointestinal function recovery during postoperative care of CRC patients (17). Research has shown that promptly initiating soluble dietary fiber for enteral nutrition offers advantages, including improved nutritional markers, gradual weight loss, and a lower incidence of gastrointestinal complications. These interventions have significantly improved nutritional wellbeing after surgery, leading to a reduction in complications. Dietary fibers are more than inert plant materials in the human digestive system; they interact actively with nutrients, generating beneficial metabolites, regulating nutrient absorption, and stimulating the growth of small intestine villi (18). This interactive process leads to increased nutrient absorption and an overall enhancement in nutritional status. Categorized as soluble or insoluble, these fibers exhibit specific traits - soluble fibers undergo fermentation easily, unlike insoluble fibers. Recent studies have highlighted the significance of colon dietary fiber fermentation, resulting in the synthesis of short-chain fatty acids (19). These fatty acids, after transportation to the liver through the portal venous system, can be transformed into glutamine, a vital nutrient that directly nourishes the small intestine through the bloodstream, enhancing nutritional absorption and overall wellbeing (20).

Enteral nutrition support containing dietary fiber can prevent the occurrence of intestinal flora disorder, effectively inhibit the reproduction of intestinal pathogenic bacteria, and promote the growth of probiotics (21, 22). The findings of this study indicated that following the intervention, the levels of PCT, *β*-EP, CRP, IL-1, IL-8, and TNF-*α* were lower in the observation group compared to the control group. Contrastingly, there was an increase in the levels of Hb, ALB, PA and TRF, particularly notable post-14 days (*p* < 0.01). This indicates that enteral nutrition support with dietary fiber can reduce the infection stress response and inflammatory factor levels of patients after laparoscopic CRC surgery, promote the improvement of the nutritional status of patients, accelerate the recovery of intestinal function (23, 24).

In response to appropriate dietary fiber intake, gastrointestinal hormones are boosted, aiding in the restoration of intestinal motility following CRC surgery (25). Studies suggest that such fiber intake heightens small intestine activity significantly. Our observations reveal shortened intervals for gas passage post-surgery in the dietary fiber group compared to controls (47.74 ± 5.23 h vs. 59.89 ± 5.11 h, *p* < 0.01). Similarly, the dietary fiber group displayed quicker recovery of intestinal sounds (25.31 ± 3.36 h vs. 34.26 ± 3.57 h, *p* < 0.01), indicative of enhanced intestinal peristalsis recovery. Inflammation signifies the body’s acute reaction to tissue injury induced by microbial infections and other harmful triggers (26). This study aims to explore dietary fiber’s impact on postoperative immune function and inflammatory responses in CRC patients. Our findings depict a notable decline in immune parameters for both patient groups by the seventh postoperative day, reflecting a potential compromise in immune functionality. However, by the 14th day, these indices showcased gradual improvement. Notably, on the 14th-day post-surgery, the observation group exhibited significantly elevated levels of CD4+, IgA, and IgG in comparison to the control group (39.21 ± 6.67 vs. 35.15 ± 6.21, 3.19 ± 0.69 vs. 2.53 ± 0.60 g/L, and 10.93 ± 3.53 vs. 9.03 ± 7.78 g/L, respectively, *p* < 0.01). This underscores the role of dietary fiber in enhancing immune responses and bolstering humoral and cellular immunity postoperatively (27, 28). Moreover, dietary fiber improves postoperative gastrointestinal motility, reducing the chances of abdominal distension and diarrhea (29). This nutritional intervention method ensures patient safety throughout the perioperative phase and promotes swift recovery.

Recently, artificial intelligence has made significant advancements in medicine (30–33). Machine learning utilizes clinical data attributes and algorithms to predict outcomes and develop models (34). Comparing different algorithms can enhance the accuracy of clinical predictions. This technology analyzes diverse data modules to identify outcome-related variables, discover risk factors, and explore patterns, facilitating the iterative refinement of mathematical models. This research seeks to develop a cost-effective and highly accurate diagnostic system for colorectal cancer CRC, with the intention of aiding clinicians in making timely and informed decisions (35). We utilized clinical outcomes from CRC patients to construct four machine learning models—LR, RF, NN, and SVM—to predict the impact of dietary fiber on postoperative immune function and inflammation. The NN model outperformed the others, achieving an AUC of 0.851 in training and 0.861 in testing. Consequently, the NN model was selected as the final model due to its superior performance.

Recently, many experts have used predictive models to assess the impact of variables on outcome indicators, achieved through variable importance scoring (36, 37). The higher the importance score of a variable, the more significant its impact on the model’s prediction results. This study visualized weights using NN model, with the level of variable importance being positively correlated with the length of the bar in the bar chart. The results revealed that the top four variables were PCT, PAB, ALB, and IL-1, indicating that these variables have a significant impact on the prediction of CRC.

## 5 Conclusion

Early postoperative intake of dietary fiber is feasible for improving the condition of CRC patients. The LR, RF, NN, and SVM models developed in this study reliably diagnosed CRC, with the NN model showing the highest accuracy. Machine learning models offer considerable clinical value in diagnosing and predicting CRC and are anticipated to serve as supplementary treatment options for patients.

## Data Availability

The raw data supporting the conclusions of this article will be made available by the authors, without undue reservation.
